# Lessons from experiences of accessing healthcare during the pandemic for remobilizing rheumatology services: a national mixed methods study

**DOI:** 10.1093/rap/rkac013

**Published:** 2022-02-16

**Authors:** LaKrista Morton, Kevin Stelfox, Marcus Beasley, Gareth T Jones, Gary J Macfarlane, Peter Murchie, John Paton, Rosemary Hollick

**Affiliations:** 1 Epidemiology Group; 2 Aberdeen Centre for Arthritis and Musculoskeletal Health, University of Aberdeen; 3 Medical Research Council Versus Arthritis Centre for Musculoskeletal Health and Work; 4 Centre of Academic Primary Care, University of Aberdeen, Aberdeen; 5 Scottish Patient Ambassador, National Rheumatoid Arthritis Society, Maidenhead, UK

**Keywords:** healthcare, COVID-19, axial spondyloarthritis, PsA, pain

## Abstract

**Objectives:**

To understand the impact of the coronavirus disease 2019 pandemic on access to healthcare services for patients with inflammatory and non-inflammatory musculoskeletal (MSK) conditions.

**Methods:**

Three established cohorts that included individuals with axial SpA, psoriatic arthritis and MSK pain completed a questionnaire between July and December 2020. In parallel, a subset of individuals participated in semistructured interviews.

**Results:**

A total of 1054 people (45% female, median age 59 years) were included in the quantitative analyses. Qualitative data included 447 free-text questionnaire responses and 23 interviews. A total of 57% of respondents had tried to access care since the start of the UK national lockdown. More than a quarter reported being unable to book any type of healthcare appointment. General practice appointments were less likely to be delayed or cancelled compared with hospital appointments. Younger age, unemployment/health-related retirement, DMARD therapy, anxiety or depression and being extremely clinically vulnerable were associated with a greater likelihood of attempting to access healthcare. People not in work, those reporting anxiety or depression and poorer quality of life were less likely to be satisfied with remotely delivered healthcare. Participants valued clear, timely and transparent care pathways across primary care and specialist services. While remote consultations were convenient for some, in-person appointments enabled physical assessment and facilitated the development and maintenance of clinical relationships with care providers.

**Conclusions:**

We identified patient factors that predict access to and satisfaction with care and aspects of care that patients value. This is important to inform remobilisation of rheumatology services to better meet the needs of patients.

Key messages

- Individuals with musculoskeletal conditions experienced difficulties accessing community and specialist healthcare services during pandemic restrictions.
- Sociodemographic and clinical factors predicted the likelihood of accessing care and satisfaction with remotely delivered healthcare.
- Patients value clear, flexible care pathways responsive to individual preferences, circumstances and care needs.

## Introduction

The coronavirus disease 2019 (COVID-19) pandemic has significantly impacted people’s normal access to healthcare and the way it is delivered to them. In the UK, the first national lockdown was declared from 23 March 2020. During the lockdown, most scheduled and elective services within secondary care were temporarily suspended and then resumed only remotely [1], while specific services were prioritized (e.g. cancer treatment). In primary care, the first few months of the lockdown saw a 30% reduction in general practice (GP) consultations across England [2, 3]. Furthermore, the rates of referrals, new prescriptions and immunizations decreased more than that expected by the reduction in consultations [2, 3]. Consultations that went ahead were mostly conducted remotely.

People with musculoskeletal (MSK) conditions access more care than those in the general population [4]. Prior to the COVID-19 pandemic, most of the care for people with MSK conditions was delivered in person across community and hospital-based services. While some MSK conditions are managed mainly in primary care with input from specialist services as required (e.g. chronic non-inflammatory conditions), care for those with inflammatory rheumatic conditions treated with immunosuppressives is led by specialist rheumatology services. During the first wave of the pandemic, pain management services, physiotherapy and elective orthopaedic services were suspended. Many staff were redeployed to frontline COVID-related care. Some people with MSK conditions, particularly those inflammatory rheumatic conditions treated with immunosuppressive therapies, were identified as clinically extremely vulnerable (CEV) and advised to ‘shield’, which included advice to stay at home and not go to work.

Some of the changes to healthcare resulting from the pandemic have been positive, others less so. Positives include enhancements in outreach primary care and mental health for vulnerable groups, online health promotion and community admission avoidance aimed at enhancing access to care and preventing unnecessary hospital attendance [5]. Recent evidence suggests there has been a decrease in emergency department attendance for non-urgent complaints [6]. Rheumatology services were advised to limit face-to-face contact with patients [7, 8] and telemedicine was identified as being well placed to deal with the challenges this presented [9–11]. ‘Attend anywhere’, a platform to support video consultations in outpatient settings was rolled out nationally in the UK in March 2020 [10]. However, suspension of elective care has led to a significant backlog in elective treatments, with waiting lists for treatment in England standing at 5.7 million in August 2021 [12], and emerging evidence that sicker people have avoided care [13]. A recent study of rheumatology patients’ and clinicians’ views on telemedicine has highlighted some issues around its acceptability and safety [14]. The COVID-19 pandemic has also exacerbated existing strains within the healthcare system, such as insufficient staffing in rheumatology services [15] and, more broadly, care capacity across community and hospital settings and a lack of joined services to help patients home from the hospital when they are ready [16].

Emerging evidence also suggests that the COVID-19 pandemic has had direct and indirect impacts on different subsets of the population. The risk of dying from COVID-19 is greater in older people, males, those living in deprived areas and ethnic minority groups [17]. However, there is also evidence of excess population mortality, in addition to deaths from COVID-19, particularly in those <75 years from ethnic minority groups, irrespective of area deprivation, and in white groups in the most deprived quintiles [18].

Moving beyond the pandemic, healthcare services have an opportunity to remobilize and reconfigure service provision and delivery for people with chronic MSK conditions in an equitable way that is shaped by and prioritizes aspects of care that patients value. While several studies have explored access to specialist rheumatology care (and less frequently primary care) in online surveys that include people with self-reported MSK conditions [19–22], to our knowledge there are no studies that have looked in detail at sociodemographic and clinical factors influencing access to primary and secondary care services in well-defined clinical cohorts that include a spectrum of inflammatory and non-inflammatory MSK conditions.

The aim of the current study was to understand the impact of the pandemic on access to primary and secondary care services and individual factors influencing this in three well-defined clinical cohorts, the perceived ability of these changes to services to meet individuals’ healthcare needs and what patients value in order to inform service remobilization.

## Methods

### Study design

We performed a mixed methods study incorporating a questionnaire, qualitative responses to a free-text questionnaire item and parallel semistructured interviews. The Consolidated Criteria for Reporting Qualitative Research (COREQ) checklist was used for reporting the qualitative methods and findings (Supplementary Data S1, available at *Rheumatology Advances in Practice* online).

#### Source of participants/data

Participants from three established cohorts took part in the CONTAIN Study and the methods of data collection for this study have been published [23]. The CONTAIN Study included individuals with axial SpA who were recruited from 83 clinical sites across the UK [British Society for Rheumatology Biologics Register Ankylosing Spondylitis (BSRBR-AS) Register] [24], people with PsA who are currently being recruited from sites across the UK [British Society for Rheumatology Psoriatic Arthritis (BSR-PsA) Register] [25] and people recruited from three Scottish health boards who had consulted with regional musculoskeletal pain and other symptoms [Maintaining Musculoskeletal Health (MAmMOTH) Study] [26]. Beginning in June 2020, when the UK was coming out of its first national lockdown, participants completed a questionnaire for the CONTAIN Study. From participants who consented to further contact, we purposively selected those for interview using maximum variation sampling based on their gender, age, employment status and nature of their MSK condition.

#### Data collection

##### Questionnaire

Within the survey we collected data about sociodemographic characteristics (deprivation status was determined using postcodes with reference to the population of England [27], Scotland [28] or Wales [29]), health-related quality of life (European Quality of Life 5-Dimensions 5-Level, with higher scores reflecting a higher quality of life [30]), anxiety and depression [Patient-Reported Outcomes Measurement Information System (PROMIS) scored 0–100, with higher scores reflecting more anxiety or depression [31]), self-reported medication use about the use of biologic or targeted synthetic DMARD (bDMARDs/tsDMARDs) and/or conventional DMARDs (cDMARD) or steroids and whether they had been identified as ‘clinically extremely vulnerable’ (CEV) and advised to shield. We asked about whether healthcare appointments including GP appointments, regular review hospital appointments, new hospital appointments, treatment sessions (e.g. physiotherapy) or ‘other’ appointments had been cancelled, delayed or went ahead as planned. Participants were also able to indicate whether they had tried but were unable to book any of these appointments, as well as whether they had decided not to try to access healthcare during the pandemic. Subsequent questions asked about any experience of virtual healthcare, including a question about the method of consultation (telephone, video, other) and about how satisfied they were with their appointment (0, very dissatisfied–10, very satisfied). Written consent was obtained within the questionnaire.

##### Qualitative data

Qualitative data collection provided the opportunity to learn about people’s experiences of accessing care during the pandemic and priorities for care moving forward. An open-ended survey question asked for individuals’ perceptions of current and future changes to healthcare:

‘*The COVID-19 pandemic has brought about rapid changes to the way health and social care services are delivered (e.g. more telephone or video consultations, and less healthcare delivered face-to-face). We would be interested to learn your views about the current and future changes to health and social care during and after the pandemic’.*

Any free-text responses to this question were included within the qualitative analysis. In addition, we carried out semistructured interviews by telephone. These explored the impact of the COVID-19 pandemic on individuals’ family/social lives, work and health. Health-related questions focused on the impact of the pandemic on access to healthcare, whether care met individual needs and future priorities for the provision and delivery of healthcare. The semistructured interview guide for these topic areas is provided in Supplementary Table S1, available at *Rheumatology Advances in Practice* online.

Interviews were conducted by an independent research fellow (K.S.) with extensive prior experience and professional interest in conducting qualitative interviews with people with lived experience of chronic pain and MSK conditions. K.S. discussed the project and reasons for doing the research with each potential participant over the phone at the time of scheduling the interview and answered any questions they had at that time. Consent was subsequently obtained prior to the interview using a written consent guide and this was audio recorded. Interviews were audio recorded and transcribed verbatim.

#### Data analysis

##### Quantitative analysis

Attempts to seek care within primary and secondary care and whether appointments were cancelled, delayed, went ahead as planned or were appointments that the individual tried but was unable to book were assessed using frequencies. Univariate and adjusted logistic regression models were used to examine factors associated with attempting to seek care from primary and secondary services, individuals’ satisfaction with remotely delivered consultations (telephone or video) and factors associated with being satisfied with remote consultation (as indicated by a score ≥6 on the 0–10 satisfaction scale). Analyses were adjusted for age, gender and level of deprivation and odds ratios (ORs) were reported with 95% CIs.

The analyses used the 14 December 2020 version of the database; all analyses were conducted using Stata SE 15.1 (StataCorp, College Station, TX, USA).

##### Qualitative analysis

The transcribed interviews and free-text responses from the questionnaire were analysed using NVivo 12 software (QSR International, Hawthorne East, VIC, Australia) to facilitate organization and coding of the data. Qualitative data were analysed thematically by K.S. and L.M., supported by R.H., and the Memo function in NVivo was used to note the development of themes and ideas by the team throughout the coding process. The data were analysed using both inductively and deductively derived coding that was informed by the interview topic guide and specifically focused on generating an understanding of how changes in care delivery impacted on individual care needs and what patients value(d) about the care they receive(d). The analysis involved familiarization with data and the initial coding, organizing codes based on similarity of their meaning and developing/reviewing themes. The analysis was discussed with all authors. Thematic and code saturation determined data saturation [32].

#### Integration of data

The quantitative questionnaire data and qualitative data (free-text responses and interviews) were collected and analysed simultaneously. The quantitative and qualitative data provide complementary insights into patterns of healthcare use and issues with accessing and receiving care during the pandemic. Findings from the questionnaire were used to quantify individuals’ reported access to healthcare during the pandemic and their satisfaction with this care, while the qualitative data aimed to provide a deeper understanding of these experiences of changes to healthcare delivery, decisions about accessing care and what they valued about the care they received, in order to inform service remobilization going forward.

#### Ethics

Ethical approval for BSRBR-AS was obtained from the National Research Ethics Service (NRES) Committee North East (County Durham and Tees Valley; reference 11/NE/0374), for BSR-PsA from West of Scotland REC 3 (reference 18/WS/0126) and for MAmMOTH from NRES Committee South West (Cornwall and Plymouth; reference 16/SW/0019). Informed consent was given by participants for the publication of material.

## Results

A total of 1054 individuals who completed the questionnaire were included in the quantitative analysis (596 from BSRBR-AS, 162 from BSR-PsA and 296 from MAmMOTH), representing 29% of those invited (27% from BSRBR-AS, 26% from BSR-PsA and 33% from MAmMOTH).

Table 1 illustrates the sociodemographic and clinical characteristics of the sample, stratified by study cohort. Around 50% of the BSRBR-AS and BSRBR-PsA cohorts were on b/tsDMARDs compared with 5% of the MAmMOTH cohort. A greater proportion of those in the BSR-PsA cohort reported taking cDMARDs.

**Table 1:** Sociodemographic characteristics of respondents to the questionnaire, stratified by cohort (*n* = 1054)

Characteristics		Study cohort		
		BSRBRAS	MAmMOTH	BSR-PsA
		(*n* = 596)	(*n* = 296)	(*n* = 162)
Age, median (IQR), years		57 (44–67)	64 (54–73)	55 (46–64)
Gender, *n* (%)	Male	392 (65.8)	118 (39.9)	67 (41.4)
	Female	203 (34.1)	177 (59.8)	95 (58.6)
	Non-binary	1 (0.2)	1 (0.3)	0 (0.0)
Employment status, *n* (%)	Full time, including students and unpaid	245 (41.1)	86 (29.1)	72 (44.4)
	Working part time	88 (14.8)	22 (11.2)	28 (17.3)
	Retired	174 (29.2)	149 (50.3)	36 (22.2)
	Unemployed, seeking work	13 (2.2)	3 (1.0)	1 (0.6)
	Unemployed because of ill health/disability	22 (3.7)	4 (1.4)	14 (8.6)
	Retired early because of ill health/disability	43 (7.2)	14 (4.7)	8 (4.9)
	Missing	11 (1.9)	7 (2.4)	3 (1.9)
Deprivation, *n* (%)	1, most deprived	61 (10.2)	15 (5.1)	19 (11.7)
	2	90 (15.1)	21 (7.1)	23 (14.2)
	3	111 (18.6)	61 (20.6)	38 (23.5)
	4	164 (27.5)	113 (38.2)	39 (24.1)
	5, least deprived	170 (28.5)	86 (29.1)	43 (26.5)
Residence, *n* (%)	Urban	435 (73.0)	181 (61.1)	121 (74.7)
	Rural	161 (27.0)	115 (38.9)	41 (25.3)
Month of completion (2020), *n* (%)	July	257 (43.1)	143 (48.3)	48 (29.6)
	August	161 (27.0)	18 (6.1)	51 (31.5)
	September	18 (3.0)	9 (3.0)	7 (4.3)
	October	149 (25.0)	119 (40.2)	55 (34.0)
	November	9 (1.5)	7 (2.4)	1 (0.6)
	December	2 (0.3)	0 (0.0)	0 (0.0)
Medications, *n* (%)	bDMARDs	234 (39.2)	7 (2.4)	51 (31.5)
	tsDMARDs	39 (6.5)	8 (2.7)	26 (16.0)
	cDMARDs	80 (13.4)	14 (4.7)	95 (58.6)
	Steroids	44 (7.4)	39 (13.2)	19 (11.7)
	Missing	17 (2.9)	10 (3.4)	8 (4.9)

A total of 602 individuals (57%) told us that they had tried to access any healthcare during the pandemic. Of these, 420 had tried to access care from a GP and 438 had tried to access a hospital appointment. More than a quarter of individuals reported that they had tried but were unable to book an appointment for any type of healthcare. Fewer people were unsuccessful in booking a hospital appointment/treatment session (15.1%) compared with a GP appointment (22.6%). However, compared with hospital appointments and treatment sessions, GP appointments were less likely to be delayed or cancelled (see Table 2).

**Table 2:** Attempts at seeking healthcare during the pandemic

Type of care	Appointment outcome	n (%)
Any healthcare (*n* = 602)	Had an appointment go ahead as planned	405 (67.3)
	Had an appointment delayed	156 (25.9)
	Had an appointment cancelled	199 (33.1)
	Tried but unable to book appointment	166 (27.6)
General practice (*n* = 420)	Had an appointment go ahead as planned	301 (71.7)
	Had an appointment delayed	42 (10.0)
	Had an appointment cancelled	39 (9.3)
	Tried but unable to book appointment	95 (22.6)
Hospital appointment/treatment session (*n* = 438)	Had an appointment go ahead as planned	217 (49.5)
	Had an appointment delayed	133 (30.4)
	Had an appointment cancelled	180 (41.1)
	Tried but unable to book appointment	66 (15.1)

Different sociodemographic and clinical factors were associated with attempting to seek primary and secondary care (Tables 3 and 4]. After adjusting for age, gender and level of deprivation, those who sought healthcare were more likely to be unemployed or retired early due to health reasons [adjusted OR (OR_adj_) primary care 1.64 (95% CI 1.05, 2.56); OR_adj_ secondary care 1.94 (1.24, 3.02)], to have been prescribed a bDMARD/tsDMARD [OR_adj_ primary care 1.45 (95% CI 1.08, 1.96); OR_adj_ secondary care 2.10 (1.56, 2.82)] or cDMARD/steroid [OR_adj_ primary care 1.44 (95% CI 1.01, 2.06); OR_adj_ secondary care 1.90 (1.33, 2.71)] or to have reported some anxiety [OR_adj_ primary care 1.58 (95% CI 1.15, 2.17); OR_adj_ secondary care 1.90 (1.35, 2.67)] or depression [OR_adj_ primary care 1.70 (95% CI 1.21, 2.38); OR_adj_ secondary care 2.07 (1.47, 2.90)]. Participants who were ≤30 years of age were more likely to have accessed primary care [OR_adj_ 2.73 (95% CI 1.15, 6.47)] while those who had shielded were more likely to have sought secondary care [OR_adj_ 1.82 (95% CI 1.39, 2.38)]. The timing of when individuals completed the survey was also associated with reporting accessing care, as those who completed the survey later in the year (October–December 2020) were more likely to report accessing primary care [OR_adj_ 1.55 (95% CI 1.15, 2.08)] while those completing the survey from August 2020 onwards were more likely to report accessing secondary care [OR_adj_ 1.52 (95% CI 1.10, 2.08)]. Individuals who reported a higher health-related quality of life were less likely to have sought care [OR_adj_ primary care 0.90 (95% CI 0.85, 0.96); OR_adj_ secondary care 0.84 (0.79, 0.89)] while those in the MAmMOTH study cohort (regional MSK pain) were less likely to have sought care from secondary services [OR_adj_ 0.57 (0.42, 0.79)].

**Table 3:** Factors associated with attempting to access primary care (*N* = 1011)

Characteristics		Attempted to access primary care, *n* (%)	OR (95% CI)	Age and gender, aOR (95% CI)	Age, gender and deprivation, aOR (95% CI)
Age (years)	≥30	15 (62.5)	**2.66 (1.14, 6.22)**	**2.48 (1.06, 5.82)**	**2.73 (1.15, 6.47)**
	31–45	93 (44.7)	1.29 (0.92, 1.81)	1.26 (0.90, 1.76)	1.30 (0.93, 1.83)
	46–64	164 (38.5)	1 [ref]	1 [ref]	1 [ref]
	65–74	100 (42.0)	1.16 (0.84, 1.60)	1.19 (0.86, 1.65)	1.20 (0.87, 1.67)
	≥75	48 (41.7)	1.14 (0.75, 1.74)	1.21 (0.79, 1.85)	1.21 (0.79, 1.84)
Gender	Male	211 (38.3)	**0.75 (0.58, 0.96)**	**0.76 (0.59, 0.98)**	0.78 (0.60, 1.01)
	Female	208 (45.4)	1 [ref]	1 [ref]	1 [ref]
Study cohort	BSRBR-AS	220 (38.5)	1 [ref]	1 [ref]	1 [ref]
	MAmMOTH	130 (45.6)	**1.34 (1.01**, **1.79)**	1.32 (0.97, 1.79)	1.29 (0.94, 1.75)
	BSR-PsA	70 (45.5)	1.33 (0.93, 1.91)	1.32 (0.91, 1.90)	1.32 (0.91, 1.91)
Deprivation	1, most deprived	35 (38.5)	0.95 (0.58, 1.54)	0.89 (0.54, 1.47)	0.89 (0.55, 1.47)
	2	49 (37.7)	0.92 (0.60, 1.41)	0.92 (0.60, 1.41)	0.92 (0.60, 1.41)
	3	91 (44.8)	1.23 (0.86, 1.78)	1.23 (0.85, 1.78)	1.23 (0.85, 1.78)
	4	133 (43.6)	1.17 (0.84, 1.63)	1.20 (0.86, 1.68)	1.20 (0.86, 1.68)
	5, least deprived	112 (39.7)	1 [ref]	1 [ref]	1 [ref]
Employment (*n* = 1007)	Full time, including students and unpaid	152 (38.7)	1 [ref]	1 [ref]	1 [ref]
	Part time	63 (43.2)	1.20 (0.82, 1.77)	1.19 (0.79, 1.78)	1.19 (0.79, 1.78)
	Retired	146 (41.5)	1.12 (0.84, 1.51)	1.27 (0.79, 2.03)	1.29 (0.80, 2.08)
	Unemployed, seeking work; unemployed/retired early due to ill health	56 (48.3)	1.48 (0.98, 2.25)	**1.61 (1.04**, **2.50)**	**1.64 (1.05**, **2.56)**
Residence	Urban	292 (41.4)	1 [ref]	1 [ref]	1 [ref]
	Rural	128 (41.8)	1.02 (0.77, 1.34)	1.02 (0.78, 1.35)	0.94 (0.70, 1.25)
Shielded (*n* = 990)	Advised and followed	148 (44.4)	1.19 (0.91, 1.55)	1.19 (0.91, 1.56)	1.20 (0.92, 1.57)
	Not advised or not followed	264 (40.2)	1 [ref]	1 [ref]	1 [ref]
Month of completion, 2020	July	162 (37.8)	1 [ref]	1 [ref]	1 [ref]
	August, September	99 (39.3)	1.07 (0.77, 1.47)	1.09 (0.79, 1.50)	1.07 (0.78, 1.48)
	October–December	159 (48.2)	**1.53 (1.15**, **2.05)**	**1.57 (1.17**, **2.11)**	**1.55 (1.15**, **2.08)**
Medication (*n* = 1007)	bDMARD or tsDMARD	146 (45.8)	**1.39 (1.05**, **1.85)**	**1.43 (1.06**, **1.93)**	**1.45 (1.08**, **1.96)**
	cDMARD or steroids only	77 (46.4)	1.43 (1.00, 2.03)	**1.44 (1.01**, **2.05)**	**1.44 (1.01**, **2.06)**
	None of the above	197 (37.7)	1 [ref]	1 [ref]	1 [ref]
PROMIS anxiety (*n* = 997)	Normal	210 (36.6)	1 [ref]	1 [ref]	1 [ref]
	Mild	110 (48.3)	**1.62 (1.18**, **2.20)**	**1.57 (1.15**, **2.15)**	**1.58 (1.15**, **2.17)**
	Moderate/severe	96 (49.2)	**1.68 (1.21**, **2.33)**	**1.64 (1.17**, **2.29)**	**1.67 (1.19**, **2.35)**
PROMIS depression (*n* = 988)	Normal	238 (38.1)	1 [ref]	1 [ref]	1 [ref]
	Mild	75 (44.4)	1.30 (0.92, 1.83)	1.27 (0.90, 1.80)	1.29 (0.91, 1.82)
	Moderate/severe	99 (51.0)	**1.69 (1.22**, **2.34)**	**1.66 (1.19**, **2.32)**	**1.70 (1.21**, **2.38)**
EQ-5D^a^^,^^b^ (*n* = 1004)		0.64 (0.23)	**0.91 (0.86**, **0.96)**	**0.91 (0.86**, **0.97)**	**0.90 (0.85**, **0.96)**

EQ-5D: European Quality of Life 5-Dimensions questionnaire; ref: reference.

a Mean (s.d.).

b OR is per 0.1 of a unit.

Values in bold indicate statistical significance.

**Table 4:** Factors associated with attempting to access secondary care (*N* = 1011)

Characteristics		Attempted to access secondary care, *n* (%)	OR (95% CI)	Age and gender, aOR (95% CI)	Age, gender and deprivation, aOR (95% CI)
Age (years)	≤30	15 (62.5)	**1.41 (1.03**, **5.64)**	2.30 (0.98, 5.39)	2.16 (0.91, 5.11)
	31–45	104 (50.0)	**1.45 (1.04**, **2.02)**	**1.42 (1.02**, **1.99)**	1.41 (1.00, 1.97)
	46–64	174 (40.8)	1 [ref]	1 [ref]	1 [ref]
	65–74	98 (41.2)	1.01 (0.73, 1.40)	1.03 (0.75, 1.43)	1.03 (0.75, 1.43)
	≥75	47 (40.9)	1.00 (0.66, 1.52)	1.04 (0.68, 1.58)	1.05 (0.69, 1.60)
Gender	Male	224 (40.7)	0.79 (0.61, 1.01)	0.83 (0.64, 1.08)	0.83 (0.64, 1.07)
	Female	213 (46.5)	1 [ref]	1 [ref]	1 [ref]
Study cohort	BSRBR-AS	260 (45.5)	1 [ref]	1 [ref]	1 [ref]
	MamMOTH	96 (33.7)	**0.61 (0.45**, **0.82)**	**0.57 (0.42**, **0.79)**	**0.57 (0.42**, **0.79)**
	BSR-PsA	82 (53.3)	1.37 (0.96, 1.95)	1.32 (0.92, 1.90)	1.31 (0.91, 1.89)
Deprivation	1, most deprived	43 (47.3)	1.13 (0.70, 1.81)	1.05 (0.65, 1.69)	1.05 (0.65, 1.69)
	2	56 (43.1)	0.95 (0.63, 1.45)	0.94 (0.62, 1.43)	0.94 (0.62, 1.43)
	3	93 (45.8)	1.06 (0.74, 1.53)	1.05 (0.73, 1.52)	1.05 (0.73, 1.52)
	4	121 (39.7)	0.83 (0.59, 1.15)	0.84 (0.60, 1.17)	0.84 (0.60, 1.17)
	5, least deprived	125 (44.3)	1 [ref]	1 [ref]	1 [ref]
Employment (*n* = 1007)	Full time, including students and unpaid	168 (42.8)	1 [ref]	1 [ref]	1 [ref]
	Part time	68 (46.6)	1.17 (0.80, 1.71)	1.19 (0.80, 1.78)	1.20 (0.80, 1.80)
	Retired	134 (38.1)	0.82 (0.61, 1.10)	0.86 (0.53, 1.38)	0.86 (0.53, 1.38)
	Unemployed, seeking work; Unemployed/retired early due to ill health	66 (56.9)	**1.77 (1.16**, **2.69)**	**1.91 (1.23**, **2.97)**	**1.94 (1.24**, **3.02)**
Residence	Urban	319 (45.2)	1 [ref]	1 [ref]	1 [ref]
	Rural	119 (38.9)	0.77 (0.59, 1.01)	0.79 (0.60, 1.05)	0.80 (0.59, 1.07)
CEV (shielded) (*n* = 990)	Advised and followed	174 (52.3)	**1.76 (1.34**, **2.30)**	**1.82 (1.39**, **2.38)**	**1.82 (1.39**, **2.38)**
	Not advised or not followed	252 (38.4)	1 [ref]	1 [ref]	1 [ref]
Month of completion, 2020	July	163 (38.0)	1 [ref]	1 [ref]	1 [ref]
	August, September	119 (47.2)	**1.46 (1.07**, **2.00)**	**1.50 (1.09**, **2.05)**	**1.52 (1.10**, **2.08)**
	October–December	156 (47.3)	**1.46 (1.09**, **1.96)**	**1.50 (1.12**, **2.02)**	**1.50 (1.12**, **2.02)**
Medication (*n* = 1007)	bDMARD or tsDMARD	169 (53.0)	**2.09 (1.57**, **2.77)**	**2.09 (1.55**, **2.81)**	**2.10 (1.56**, **2.82)**
	cDMARD or steroids only	84 (50.6)	**1.90 (1.33**, **2.70)**	**1.92 (1.34**, **2.73)**	**1.90 (1.33**, **2.71)**
	None of the above	183 (35.1)	1 [ref]	1 [ref]	1 [ref]
PROMIS anxiety (*n* = 997)	Normal	213 (37.1)	1 [ref]	1 [ref]	1 [ref]
	Mild	115 (50.4)	**1.72 (1.27**, **2.35)**	**1.65 (1.21**, **2.26)**	**1.67 (1.21**, **2.28)**
	Moderate/severe	105 (53.8)	**1.98 (1.42**, **2.75)**	**1.90 (1.36**, **2.65)**	**1.90 (1.35**, **2.67)**
PROMIS depression (*n* = 988)	Normal	235 (37.6)	1 [ref]	1 [ref]	1 [ref]
	Mild	82 (48.5)	**1.56 (1.11**, **2.20)**	**1.53 (1.08**, **2.16)**	**1.55 (1.09**, **2.19)**
	Moderate/severe	110 (56.7)	**2.17 (1.57**, **3.01)**	**2.07 (1.48**, **2.90)**	**2.07 (1.47**, **2.90)**
EQ-5D^a^^,^^b^ (*n* = 1004)		0.62 (0.24)	**0.84 (0.79**, **0.89)**	**0.84 (0.79**, **0.89)**	**0.84 (0.79**, **0.89)**

EQ-5D: European Quality of Life 5-Dimensions questionnaire; ref: reference.

a Mean (s.d.).

b OR is per 0.1 of a unit.

Values in bold indicate statistical significance.

Different sociodemographic and clinical factors were also associated with deciding not to seek healthcare (Table 5). Factors associated with deciding not to seek care, after adjustment for age, gender and level of deprivation, included having moderate/severe anxiety [OR_adj_ 1.83 (95% CI 1.23, 2.70)] or depression [OR_adj_ 1.50 (95% CI 1.01, 2.24)], being in the MAmMOTH study cohort [regional MSK pain; OR_adj_ 1.56 (95% CI 1.08, 2.24)] and completing the survey during August/September [OR_adj_ 1.80 (95% CI 1.24, 2.61)]. Those who had higher health-related quality of life were less likely to avoid seeking healthcare [OR_adj_ 0.93 (95% CI 0.87, 1.00)].

**Table 5:** Factors associated with avoiding seeking healthcare (*N* = 997)

Characteristics		Decided not to try to access healthcare, *n* (%)	OR (95% CI)	Age and gender, aOR (95% CI)	Age, gender and deprivation, aOR (95% CI)
Age (years)	≤30	4 (17.4)	0.74 (0.25, 2.22)	0.73 (0.24, 2.19)	0.73 (0.24, 2.23)
	31–45	39 (18.9)	0.82 (0.54, 1.24)	0.81 (0.53, 1.24)	0.80 (0.53, 1.23)
	46–64	93 (22.2)	1 [ref]	1 [ref]	1 [ref]
	65–74	44 (19.0)	0.82 (0.55, 1.23)	0.83 (0.55, 1.24)	0.83 (0.56, 1.25)
	≥75	32 (27.4)	1.32 (0.83, 2.11)	1.33 (0.83, 2.14)	1.35 (0.84, 2.16)
Gender	Male	113 (20.8)	0.98 (0.72, 1.33)	0.94 (0.69, 1.29)	0.95 (0.70, 1.30)
	Female	97 (21.5)	1 [ref]	1 [ref]	1 [ref]
Previous study	BSRBR-AS	105 (18.9)	1 [ref]	1 [ref]	1 [ref]
	MAmMOTH	75 (26.5)	**1.57 (1.12**, **2.21)**	**1.56 (1.08**, **2.24)**	**1.56 (1.08**, **2.24)**
	BSRBR-PsA	32 (21.2)	1.17 (0.75, 1.83)	1.19 (0.76, 1.87)	1.18 (0.75, 1.86)
Deprivation	1, most deprived	24 (26.4)	1.62 (0.93, 2.83)	1.71 (0.98, 3.01)	1.71 (0.98, 3.01)
	2	23 (18.5)	1.03 (0.60, 1.78)	1.05 (0.61, 1.82)	1.05 (0.61, 1.82)
	3	42 (21.0)	1.20 (0.76, 1.90)	1.20 (0.76, 1.91)	1.20 (0.76, 1.91)
	4	72 (24.0)	1.43 (0.96, 2.14)	1.40 (0.93, 2.10)	1.40 (0.93, 2.10)
	5, least deprived	51 (18.1)	1 [ref]	1 [ref]	1 [ref]
Employment (*n* = 993)	Full time, including students and unpaid	74 (20.7)	1 [ref]	1 [ref]	1 [ref]
	Part time	28 (19.3)	0.92 (0.57, 1.49)	0.87 (0.52, 1.44)	0.85 (0.51, 1.42)
	Retired	75 (21.5)	1.05 (0.73, 1.51)	0.96 (0.54, 1.71)	0.96 (0.54, 1.72)
	Other	34 (24.1)	1.22 (0.77, 1.94)	1.16 (0.72, 1.88)	1.13 (0.70, 1.83)
Residence	Urban	140 (20.1)	1 [Ref]	1 [Ref]	1 [Ref]
	Rural	72 (24.0)	1.26 (0.91, 1.74)	1.22 (0.88, 1.70)	1.21 (0.86, 1.72)
CEV (*n* = 977)	Advised and followed	66 (19.9)	0.88 (0.64, 1.23)	0.86 (0.62, 1.20)	0.85 (0.61, 1.18)
	Not advised or not followed	142 (22.0)	1 [ref]		1 [ref]
Month of completion, 2020	July	78 (18.4)	1 [ref]	1 [ref]	1 [ref]
	August, September	72 (28.9)	**1.80 (1.25**, **2.60)**	**1.80 (1.25**, **2.61)**	**1.80 (1.24**, **2.61)**
	October–December	62 (19.1)	1.04 (0.72, 1.51)	1.02 (0.70, 1.47)	1.00 (0.69, 1.45)
Medication (*n* = 992)	bDMARD or tsDMARD	64 (20.2)	0.87 (0.62, 1.22)	0.90 (0.63, 1.29)	0.91 (0.64, 1.30)
	cDMARD or steroids only	30 (18.5)	0.78 (0.50, 1.22)	0.77 (0.49, 1.20)	0.79 (0.50, 1.23)
	None of the above	116 (22.6)	1 [ref]	1 [ref]	1 [ref]
PROMIS anxiety (*n* = 984)	Normal	108 (19.0)	1 [ref]	1 [ref]	1 [ref]
	Mild	46 (20.4)	1.09 (0.74, 1.61)	1.13 (0.77, 1.68)	1.11 (0.75, 1.64)
	Moderate/Severe	56 (29.3)	**1.77 (1.21**, **2.57)**	**1.87 (1.27**, **2.74)**	**1.83 (1.23**, **2.70)**
PROMIS depression (*n* = 975)	Normal	120 (19.4)	1 [ref]	1 [ref]	1 [ref]
	Mild	35 (21.0)	1.10 (0.72, 1.68)	1.13 (0.74, 1.73)	1.13 (0.74, 1.74)
	Moderate/Severe	49 (25.8)	1.44 (0.99, 2.11)	**1.56 (1.05**, **2.32)**	**1.50 (1.01**, **2.24)**
EQ-5D^a^^,^^b^ (*n* = 990)	Decided not to try to access	0.64 (0.22)	**0.93 (0.87**, **0.99)**	**0.93 (0.87**, **0.99)**	0.93 (0.87, 1.00)
	Did not	0.68 (0.22)			

EQ-5D: European Quality of Life 5-Dimensions questionnaire; ref: reference.

a Mean (s.d.).

b OR is per 0.1 of a unit.

Values in bold indicate statistical significance.

A total of 599 of 602 individuals who had experienced remotely delivered healthcare (telephone/video) indicated whether they were satisfied with the care they received. While individuals were generally satisfied {median score 8 [interquartile range (IQR) 6–10]}, 22% were either not satisfied or were neutral about their appointment (as indicated by a score of 0–5). Factors associated with being satisfied are provided in Table 6. After adjusting for age, gender and level of deprivation, those who were retired [OR_adj_ 0.32 (95% CI 0.15, 0.67)] or unemployed [OR_adj_ 0.38 (0.20, 0.73)], who reported mild [OR_adj_ 0.45 (0.28, 0.73)] or moderate/severe anxiety [OR_adj_ 0.58 (0.34, 1.00)] or moderate/severe depression [OR_adj_ 0.45 (0.27, 0.74)] were less likely to be satisfied with remotely delivered care. There were no differences in satisfaction with remote healthcare consultations between study cohorts. Those with higher health-related quality of life were generally more likely to be satisfied with care delivered remotely [OR_adj_ 1.10 (95% CI 1.01, 1.21)].

**Table 6:** Factors associated with satisfaction with remote healthcare consultation (*N* = 599)

Characteristics		Satisfied with remote consult, * n* (%)	OR (95% CI)	Age and gender, aOR (95% CI)	Age, gender and deprivation, aOR (95% CI)
Age (years)	≤30	12 (63.2)	0.48 (0.18, 1.27)	0.45 (0.17, 1.21)	0.40 (0.15, 1.11)
	31–45	108 (78.8)	1.04 (0.62, 1.73)	1.01 (0.60, 1.69)	1.00 (0.59, 1.67)
	46–64	187 (78.2)	1 [ref]	1 [ref]	1 [ref]
	65–74	112 (81.2)	1.20 (0.71, 2.03)	1.22 (0.72, 2.08)	1.23 (0.72, 2.08)
	≥75	48 (72.7)	0.74 (0.40, 1.38)	0.76 (0.41, 1.42)	0.76 (0.40, 1.43)
Gender	Male	224 (76.7)	0.86 (0.58, 1.26)	0.84 (0.56, 1.25)	0.84 (0.57, 1.26)
	Female	242 (79.3)	1 [ref]	1 [ref]	1 [ref]
Study cohort	BSRBR-AS	257 (77.2)	1 [ref]	1 [ref]	1 [ref]
	MAmMOTH	121 (77.6)	1.02 (0.65, 1.61)	0.96 (0.59, 1.58)	0.96 (0.58, 1.58)
	BSR-PsA	89 (80.9)	1.25 (0.73, 2.15)	1.17 (0.67, 2.04)	1.12 (0.65, 1.96)
Deprivation	1, most	39 (84.8)	1.63 (0.68, 3.92)	1.78 (0.72, 4.39)	1.78 (0.72, 4.39)
	2	59 (74.7)	0.86 (0.46, 1.60)	0.85 (0.46, 1.59)	0.85 (0.46, 1.59)
	3	100 (80.0)	1.17 (0.67, 2.05)	1.16 (0.66, 2.04)	1.16 (0.66, 2.04)
	4	132 (76.7)	0.97 (0.58, 1.59)	0.90 (0.54, 1.50)	0.90 (0.54, 1.50)
	5, least	137 (77.4)	1 [ref]	1 [ref]	1 [ref]
Employment (*n* = 597)	Full time, including students and unpaid	178 (80.9)	1 [ref]	1 [ref]	1 [ref]
	Part time	85 (84.2)	1.25 (0.67, 2.36)	0.99 (0.51, 1.94)	0.98 (0.5, 1.92)
	Retired	152 (74.9)	0.70 (0.44, 1.12)	0.33 (0.16, 0.68)	0.32 (0.15, 0.67)
	Unemployed, seeking work; unemployed/retired early due to ill health	51 (69.9)	**0.55 (0.30**, **0.999)**	**0.41 (0.22**, **0.79)**	**0.38 (0.20**, **0.73)**
Residence	Urban	326 (76.9)	1 [ref]	1 [ref]	1 [ref]
	Rural	141 (80.6)	1.25 (0.81, 1.93)	1.24 (0.79, 1.93)	1.31 (0.82, 2.09)
Shielding (*n* = 586)	Advised and followed	181 (79.7)	1.18 (0.79, 1.78)	1.17 (0.77, 1.76)	1.13 (0.75, 1.71)
	Not advised/not followed	276 (76.9)	1 [ref]	1 [ref]	1 [ref]
Month of completion, 2020	July	188 (78.7)	1 [ref]	1 [ref]	1 [ref]
	August, September	123 (76.9)	0.90 (0.56, 1.46)	0.86 (0.53, 1.41)	0.88 (0.54, 1.43)
	October–December	156 (78.0)	0.96 (0.61, 1.52)	0.95 (0.60, 1.51)	0.94 (0.59, 1.50)
Medication (*n* = 597)	bDMARD or tsDMARD	172 (78.2)	1.07 (0.70, 1.65)	1.16 (0.74, 1.83)	1.18 (0.74, 1.87)
	cDMARD or steroids only	87 (80.6)	1.24 (0.71, 2.16)	1.27 (0.73, 2.23)	1.26 (0.72, 2.22)
	None of the above	207 (77.0)	1 [ref]	1 [ref]	1 [ref]
PROMIS anxiety (*n* = 590)	Normal	262 (82.9)	1 [ref]	1 [ref]	1 [ref]
	Mild	108 (70.1)	**0.48 (0.31**, **0.76)**	**0.48 (0.30**, **0.77)**	**0.45 (0.28**, **0.73)**
	Moderate/severe	91 (75.8)	0.65 (0.39, 1.08)	0.63 (0.37, 1.07)	0.58 (0.34, 1.00)
PROMIS depression (*n* = 583)	Normal	291 (82.0)	1 [ref]	1 [ref]	1 [ref]
	Mild	78 (74.3)	0.64 (0.38, 1.06)	0.62 (0.37, 1.04)	0.62 (0.37, 1.05)
	Moderate/severe	86 (69.9)	**0.51 (0.32**, **0.82)**	**0.49 (0.30**, **0.80)**	**0.45 (0.27**, **0.74)**
EQ-5D^a^^,^^b^ (*n* = 594)		0.66 (0.21)	**1.10 (1.01**, **1.20)**	**1.09 (1.00**, **1.19)**	**1.10 (1.01**, **1.21)**

EQ-5D: European Quality of Life 5-Dimensions questionnaire; ref: reference.

a Mean (s.d.).

b OR is per 0.1 of a unit.

Values in bold indicate statistical significance.

## Patients’ experiences and priorities for care

We included 447 responses to the free-text questionnaire item in the qualitative thematic analysis. Of the respondents, 54.4% were from BSRBR-AS, 14.1% from BSR-PsA and 31.5% from MAmMOTH; 52.6% were female and had a median age of 60 years (range 21–92).

Of 782 questionnaire respondents who agreed to be approached about an interview, 23 participants with axial SpA (*n* = 8), PsA (*n* = 9) and chronic pain (*n* = 6) were selected for a one-off telephone interview and consented for this. Of those, 10 were female and 12 had been advised to shield. Interviewees ranged in age from 28 to 86 years and lived in England (*n* = 13), Scotland (*n* = 8) and Wales (*n* = 2). The median duration of each interview was 39 min (range 21–53).

Key emergent themes identified within qualitative interviews and free-text questionnaire responses relating to individuals’ experiences of care and perceptions of delivery of care in the future are discussed below and further illustrative quotes are provided in Supplementary Table S2, available at *Rheumatology Advances in Practice* online. Following each quote, ‘Q’ indicates that a quote was taken from a participant’s free-text questionnaire response and ‘I’ indicates that the quote is from an interview.

## Theme 1: communication and relationships with healthcare professionals

The survey identified that individuals who reported higher levels of anxiety were more likely to attempt to access primary [OR_adj_ 1.67 (95% CI 1.19, 2.35)] and secondary care [OR_adj_ 1.90 (95% CI 1.35, 2.67)]. Individuals who reported mild anxiety were also less satisfied with remotely delivered care [OR_adj_ 0.45 (95% CI 0.28, 0.73)]. However, the survey also indicated that people with higher levels of anxiety were more likely to avoid accessing care [OR_adj_ 1.83 (95% CI 1.23, 2.70)]. The interviews provided some insights into this, illustrated in the quotes below. Some who were anxious were keen to seek reassurance from their GP. However, perceptions about inaccessibility of care and concerns about the mode of communication (not being able to ‘see’ a healthcare professional) contributed to anxiety about their symptoms/condition and sometimes led to decisions to avoid seeking care.

*I hated feeling that I couldn't go to see my GP for reassurance about my health, as I didn't want to bother them and they would only talk to you on the telephone anyway or ask you to e-mail them photos. This led to anxiety about whether symptoms were important or not. I think you need to see GPs face-to-face. I wanted to see the physiotherapist at my GP practice, but there would be no point in doing that over the phone so I left it*. Q3092 (Female, age 46–64 years, MAmMOTH)

Patients’ experiences of accessing healthcare during the pandemic illustrated the importance of communication in developing or maintaining relationships with healthcare professionals and its impact on care. The mode of care delivery (i.e. face-to-face *vs* remote) impacted on perceptions of these relationships and on the perceived quality of care received.

*Just before lockdown I met my new rheumatologist which I was meant to see three, four times since March but I haven’t. I was meant to change treatment in preparation for a baby next year but this is now 6 months behind and only spoken once on the phone to check if I was ok. I know they are trying their best but we live day in day out struggling to keep moving forward. When there’s a delay like this you have to start all over again to get yourself back on track.* Q1666 (Female, <30 years, BSRBR-AS)

*I think you feel more remote from the people caring for you…I think there’s a lack of communication that doesn’t fill you with confidence that you are being cared for, or that there’s anyone out there who cares about you!* I15 (Male, 46–64 years, BSRBR-AS)

Having previously established relationships with healthcare professionals, and previous experience of remote consultations, helped patients to feel more confident in remotely delivered care.

*It’s quicker, and it’s a lot more concise and I know the people that I’m talking to so I can quite easily talk to them over the phone as I could face to face and I’ve only had to literally go into hospital for vital tests and things that I needed to do and I just feel that that’s got to be better for everybody, you know, for me and for the hospital and for the NHS.* I11 (Female, 46–64 years, BSRBR-AS)

However, remotely delivered care was sometimes described as a means of care that was distinct from (i.e. lesser than) a face-to-face consultation. Some patients’ concerns about remotely delivered consultations illustrate the importance of face-to-face examination and discussion. Remote consultations were sometimes perceived to be less reassuring and less effective for new issues, issues that required physical examination or for instances in which there may be other concerns requiring discussion.

*Over the phone you lose the personal side of the appointment. When you are face to face, sometimes a throwaway comment which you yourself doesn’t believe is important will come out during a conversation and it is also easier to explain what’s going on.* Q2093 (Female, 46–64 years, BSR-PsA)

Some described a willingness to ‘do anything’ in order to see their healthcare professional face-to-face, emphasizing the importance of mode of delivery in maintaining clinical relationships.

*I’d go in in full PPE if I had to, you know, anti-bac every2seconds, I’d wash my hands, I’d wash my clothes when I get in. I’d do anything. I’d rather do that and see my nurse face to face so I can actually talk to her and she can see my emotions, rather than having the telephone conversation.* I8 (Female, ≤30 years, BSRBR-AS)

## Theme 2: transparent, timely and effective pathways of care

More than 25% of participants in our questionnaire sample were unable to access any care. A common challenge described by many participants in our qualitative sample was knowing who to contact and when for new or worsening symptoms. While access to rheumatology and other specialist services was important, primary care was often the first point of contact for patients with MSK conditions. A lack of clarity about what type of care could be expected from whom was frustrating and could lead to more anxiety and delays in accessing care.

*On more than one occasion I felt I would have liked to have seen a GP but instead I was either told to just take antibiotics or was actually fobbed off and told to find another clinic to deal with the problem I was having.* Q2083 (Male, 31–45 years, BSR-PsA)

*I’m sitting on my own condition, wondering whether I need to see a dermatologist, I’ve got a bit of stress that I may not be able to be seen.* I15 (Male, 46–64 years, BSRBR-AS)

Clear systems of communication and timely means of accessing care were important.

*My health teams at the hospital seem to have embraced technology and have kept in touch throughout by any means available to them. The GP practice however is a different story I am afraid, and have gone from unhelpful to obstructive using technology to hide behind. Even a phone appointment is not easily obtained now and a phone enquiry can see you waiting on the phone for 25 minutesbefore being answered and then informed that no appointments are available, please try later or tomorrow.* Q1450 (Male, 65–74 years, BSRBR-AS)

The survey found that people on biologics were more likely to attempt to access primary [OR_adj_ 1.45 (95% CI 1.08, 1.96)] and secondary care [OR_adj_ 2.10 (95% CI 1.56, 2.82)]. The interviews identified that people recognized the importance of ongoing blood monitoring for DMARD therapy and other clinical assessments for effective management of their condition. While some were anxious about attending healthcare settings in person, lack of monitoring was also a cause for anxiety.

*I needn’t really have been as anxious as I was, just inside the door they had a desk where they gave you a mask and some hand sanitiser and then it’s not far from the entrance actually to the blood department, and there were hardly any people about at all and everyone was masked so it wasn’t very worrying at all when it came to it.* I3 (Female, ≥75 years, BSR-PsA)

*Am concerned my bloods aren’t being monitored so I’m concerned we won’t be able to manage my condition as effectively as we have….* Q1692 (Female, 46–64 years, BSRBR-AS)

Moving forward, participants highlighted the importance of flexibility and of having service pathways that are responsive to individual preferences and circumstances and that allow for shared decision making about the mode of service delivery.

*Technology has jumped forward5years at least, if embraced, I think healthcare could really improve the efficiency of its service. Mandatory/recurring appointments by phone couldminimizewaiting on clinical appointments that weren’t necessary… prior to this year my 6 monthly/annual appointments have been‘how are you?’‘I'm fine’type affairs. It’s taken at least3hoursout of my day, and the same from healthcare professionals…Imagine the improvement in care for those patients that really need far more time, there’s so many possibilities.* Q1476 (Male, 31–45 years, BSRBR-AS)

## Theme 3: equitable access to care for everyone

The survey did not find that older individuals were overall less satisfied with remotely delivered care [OR_adj_ 0.76 (95% CI 0.40, 1.43)]. However, the qualitative data revealed specific challenges around adopting new technology for older individuals who may require additional support.

*I do not use video, have no mobile phone and on the rare time I consult a GP, examination is necessary. My husband has hearing problems, so even phone appointments may not work.* Q3038 (Female, ≥75 years, MamMOTH)

Individuals sometimes acknowledged that changes in the mode of care delivery required adaptation and a period of adjustment, but that there could be benefits.

*I mean, I’m 73 and I’ve been used to face to face with doctors for 65 years, so that’s a massive change. A lot of people are more reticent on the phone; a lot of people are more outgoing on the phone, so how do they balance that?* I5 (Male, 65–74 years, BSRBR-AS)

*Where possible, video/telephone interviews could be used as a means to do regular calls to certain groups e.g. over-80sor people with long-term conditions.* Q3312 (Female, ≥75 years, MamMOTH)

The qualitative data also illustrated that patients valued a service that could meet the needs of others—not just their own. Some highlighted concerns that an emphasis on remotely delivered services may present challenges for those who are older, socially isolated or are without the skills and/or finances to use technology.

*I also think increased tele appointments with GPs is a good thing, but we must have good social care in the community to support those vulnerable people who may have attended GP surgeries unnecessarily for reasons of isolation or other social needs.* Q1417 (Male, 46–64 years, BSRBR-AS)

*I’m ok, [it’s] the generation one above me. I think they struggle with just holding the technology in the right place and that actually causes a lot of stress from worrying about will they get in, won’t they get in, can they remember the code. So I think for some people, yes it’s a lot,but in my case, no impact because that GP thing has actually been more convenient.* I10 (Male, 46–64 years, BSR-PsA)

Broader concerns included an awareness that not everyone may be able to recognize when and where to access care, that self-management is a skill that must be learned and that services should be responsive to the specific needs of those with complex conditions.

*I am a former healthcare professional and generally confident on the phone—Irealizethese consultations may be trickier for some people.* Q3176 (Female, 65–74 years, MAmMOTH)

*Current arrangements do not support people with complex conditions. Don’t think that telephone appointments work,as MSK conditions are not just measured by pain.* Q2152 (Male, 46–64 years, BSR-PsA)

However, some changes in care delivery made during the pandemic were helpful in addressing, for example, geographical inequalities in access to care.

*So I think blurring the boundaries of geographical location, I think it’s really helpful in some cases. So many people can’t get about that easily, they need to get an ambulance,it’s complicated where actually a lot of things could happen online.* I23 (Female, 31–45 years, MAmMOTH)

*I live in Village - 5 miles from health centre in Town. Poor bus service, taxi £13/15 each way. Absolute priority for health centre is an updated, efficient & fully staffed telephone system*. Q3308 (Male, ≥75 years, MAmMOTH)

Taken together in the context of the national COVID-19 public health measures, individual sociodemographic and clinical factors and the themes emerging from the qualitative data interacted to influence—positively or negatively—individuals’ experiences and decision making in relation to accessing and receiving healthcare.

*I had a telephone consult with the chronic pain team, but was advised that my steroid injection would be postponed until a vaccine was in place for COVID-19. I may get some other treatment, but likely to be 2021. This was really disappointing,as I am really suffering at the moment. This was done by telephone and my GP did all the communicating by letter. Very efficiently* [Theme 2: expectations about care delivery]. *The GP I currently see has always been good about communicating by e-mail or phone for my ongoing issues that maybe don’t always require an appointment every time* [Theme 1: established relationships; mode of delivery]*. I live 40 minutesdrive from the surgery so she is sympathetic to this knowing I have trouble sitting for long periods. I am very lucky in this respect* [Theme 3: individual challenges to accessing care]*.* Q3039 (Female, 46–64 years, MAmMOTH)

## Discussion

This mixed-methods study has shown that more than a quarter of people with MSK conditions and symptoms who attempted to access any healthcare during the period of public health restrictions in the UK due to COVID-19 were unable to do so. Compared with hospital appointments and treatment sessions, GP appointments were less likely to be delayed or cancelled. Factors associated with a greater likelihood of attempting to access healthcare included not being in work, DMARD therapy, anxiety or depression and being classified as clinically extremely vulnerable (shielding). Most participants reported satisfaction with remote consultations, although people not in work, those reporting anxiety or depression and those reporting a poorer quality of life were less likely to be satisfied with remotely delivered healthcare. Interviews highlighted the importance of a balance between convenience *vs* the need to develop and maintain clinical relationships and a visual/hands-on approach to care. Primary care remains an important point of contact for people with MSK conditions and participants valued transparent and clearly defined care pathways between primary and secondary care.

Our study included people with both inflammatory and non-inflammatory MSK conditions in well-characterized cohorts of real-world patients [33], pre-defined by symptoms or a clinician-confirmed diagnosis as opposed to convenience samples. Much of the literature to date on the impact of the COVID-19 pandemic on individuals with rheumatic and MSK conditions has focused on the perceived risks of COVID-19 and immunosuppressive medication, decisions to stop/continue medication, access to medication and disruptions to specialist healthcare services [21, 34, 35]. We specifically explored access to primary as well as secondary care–based services. A number of internet surveys were conducted during the pandemic and our patient partners commented on ‘survey fatigue’ among patients, which may explain the relatively low participation rates in the current survey. We only have data on a broad perception of ‘satisfaction of care.’ We do not know which aspects of satisfaction were considered when responding to this question; e.g. satisfaction with mode of delivery, outcome or both.

The 6 month period over which study data were collected represented different degrees of COVID-19 public health measures, which not only varied over time but also depending on where people lived across the UK. A UK-wide lockdown was announced 23 March 2020; restrictions began to ease to differing extents across the devolved nations from May 2020 and shielding ended across the UK on 1 August 2020. Remobilization of secondary care services began in August 2020, including release of staff from COVID-19 work and the resumption of elective care. While primary care services remained open throughout the lockdown period, care was delivered in a very different way, with telephone triage and fewer in-person appointments. Following the end of the UK-wide lockdown, tiered systems of restrictions were introduced in August 2020 that differed across Scotland, England and Wales, with a series of local/regional lockdowns in September and October 2020 before England re-entered a short period of national lockdown on 5 November 2020 followed by a further tightening of restrictions across the UK in December 2020 [36].

These changing public health restrictions were reflected in the survey data where, compared with participants completing the survey in July 2020 (when shielding and several national lockdown restrictions were still in place across the UK), those completing the survey from August 2020 onwards were more likely to report accessing secondary care, while those completing the survey later in the year (October–December 2020) were more likely to report accessing primary care. The greater likelihood of reporting access to primary care later in 2020 may reflect perceptions that primary care had ‘shut down’ or was inaccessible, and consequently some people perhaps delayed/postponed consulting when they felt they could wait. Similarly, for those who reported delaying seeking access to care, they were more likely to have completed the survey in August/September 2020, which may reflect ongoing anxiety about COVID-19 despite healthcare services opening up again and a decision to postpone consulting.

During the course of these public health restrictions, we have shown that individuals with MSK conditions experienced difficulties accessing both community and specialist healthcare services. Most studies to date exploring access to healthcare have focused on access to rheumatology specialist services [19, 34, 35, 37, 38]. In keeping with other studies, we found that the COVID-19 pandemic led to reduced access to specialist care for people with MSK conditions [19, 35, 39]. However, we have also shown that primary care remains an important first point of contact for many people with long-term MSK conditions. While almost one-quarter of respondents reported having tried and failed to access primary care, such appointments were still more likely to go ahead and were less likely to be cancelled than secondary care appointments.

We have identified clinical and sociodemographic factors that were associated with accessing care and satisfaction with remotely delivered healthcare. We have shown that those on b/tsDMARDs were more likely to attempt to access secondary-based care than those who were not, whether or not those attempts were successful. There has been some evidence to suggest that clinically extremely vulnerable patients were less likely to access care due to concerns about the risk of COVID-19 infection or overburdening staff [37, 39]. Our results may reflect the different healthcare contexts in which they were undertaken, e.g. insurance-based systems in the USA *vs* public healthcare systems in the UK, as well as the study timing, as public health restrictions, COVID-19 levels within the population, perceptions of risk, and access to healthcare services varied over time. Individuals not in work, those reporting anxiety or depression and those reporting poorer quality of life were more likely to attempt to access healthcare and were less likely to be satisfied with remotely delivered healthcare. In this study, deprivation did not confound these relationships. These findings may reflect differences in clinical need and the ability to engage in remote consultations as well as their perceived effectiveness for a given problem. Such individuals may have greater overall debility and fewer resources to effectively access remote delivered healthcare, as illustrated by Wherton *et al.* [40] in a study of video consultations pre and during the COVID-19 pandemic in Scotland. They found that comorbidities and pre-existing conditions, along with a general level of debility influenced people’s ability to use video technology. Additional factors included low digital literacy, access to appropriate devices, internet connectivity and lack of a private space at home.

Around one-fifth of participants were not satisfied with remote consultations and interviews revealed mixed views as to the role of remote healthcare consultations. Many people felt that while remote consultations were useful for some things (e.g. issues that were perceived to be simple or straightforward), they were concerned that certain important clinical issues may be missed, such as physical signs. Others reported that remote care delivery minimized the space to divulge certain personal and social issues. Furthermore, it was not always easy to articulate complex problems by telephone. Our study findings are in keeping with Hewitt *et al*. [41], who found that telephone consultations offered fewer opportunities for disclosure of other concomitant issues and GPs were less likely to question individuals’ ideas about possible diagnoses. We found that patients valued face-to-face consultations for complex conditions or new symptoms. Similarly, Wherton et al. [40] found that healthcare providers also perceived a number of conditions unsuitable for remote consultations, i.e. where visual examination was crucial, unpredictable conditions and rarer conditions.

We also identified factors related to deciding to avoid seeking healthcare. While those who had greater anxiety and depression were more likely to attempt to seek care, they were also more likely to avoid seeking care. The qualitative data illustrated that these relationships could be due to a desire for reassurance about their symptoms/condition, but also anxiety about attending appointments in healthcare settings and about whether they would be able to access the care they perceived they needed. Individuals from the chronic pain cohort were also more likely to avoid attempting to access care—perhaps as a result of a perception that there was less that could be done for pain management at the time [42–44]. Sloan *et al*. [45] further explored aspects of satisfaction and have identified relationships between patient-reported trust and satisfaction with care and multiple patient behaviours such as reporting all symptoms to a doctor and adherence to medical advice.

On a background of COVID-19 public health measures, our quantitative and qualitative findings together suggest a complex interplay of sociodemographic, clinical and other factors relating to communication and relationships with healthcare professions, pathways of care and equitable access to care influencing experiences and decision making in relation to accessing and receiving healthcare.

Moving forward, patients valued clear, flexible care pathways responsive to individual preferences, circumstances and care needs. Experiences with remote consultations highlighted the parts of communication that could not be replaced with technology. In particular, clinical relationships were highly valued and remote consultations could make that difficult, especially if someone does not have an established relationship with a care provider. Our study findings agreed with Wherton *et al*. [40], who found that perceptions on how remote consulting altered the relationship and interaction between patients and clinicians was highly contingent upon the clinician’s interaction styles, perceived value of tactile information and patients’ facial expression and the clinical context of the encounter. Feeling supported by all care providers was also important, and primary care remained an important first point of contact. This is in keeping with Sloan et al. [39], who demonstrated that feeling medically supported was positively associated with well-being scores, the importance of ‘checking in’ with services. Those patients with pre-existing trusted medical relationships expressed less anxiety and more confidence that support would be there if required.

In summary, this study provides evidence of difficulties accessing healthcare for those with MSK conditions and symptoms during restrictions resulting from the COVID-19 pandemic. We have identified clinical and social factors associated with a greater likelihood of attempting to access care and those who were less likely to be satisfied with remote consultations. It has also provided insights into what patients value most about their care. This offers valuable lessons to enable us to target services to better meet the needs of people with MSK conditions and to develop services that are aligned with patient values and preferences.

## Supplementary Material

rkac013_Supplementary_Data
