# Supplementary material for: Lessons from experiences of accessing healthcare during the pandemic for remobilizing rheumatology services: a national mixed methods study
Source: Rheumatol Adv Pract. 2022 Feb 16;6(1):rkac013. doi: 10.1093/rap/rkac013 (PMC8946474; doi:10.1093/rap/rkac013)
Supplement: rkac013_Supplementary_Data [file rkac013_supplementary_data.zip › 21-174 Supplementary Tables.docx]

| **Supplementary Table S1.** Semi-structured interview topic guide for healthcare-related questions | |
| --- | --- |
| **Health/social care and medical treatments** | |
| **Access to health and social care services**  *Understand how patient’s access to care and treatment has been affected by pandemic* | - Could you describe how your healthcare has been affected by the pandemic, for example have any appointments been cancelled or treatments delayed? - Have you had any virtual appointments, online or by telephone? How have you found these? - How has your ability to get medications changed, if at all? - How concerned are you about having to attend hospital/GP practice for appointments/receive treatments/undergo tests during the pandemic or in the future? - Have you had any other health conditions or other health concerns during the pandemic that you feel need medical attention/advice? - Have you sought advice or support from non-NHS services during the pandemic? |
| **Influence of COVID-19 on treatment decisions**  *Understand how patient’s treatment decisions have been impacted by COVID-19 (for current or future treatments)* | - Have any of your concerns about the COVID-19 pandemic influenced decisions to start new medications for your MSK condition, or to stop existing medication (e.g. biologic therapy)? - How do think the COVID-19 pandemic will influence any future decisions about starting new treatments (e.g. immunosuppressive therapy)? |
| **Changes to health and care services in the future** | |
| **Delivery of future services**  *Understand patient’s perspective of how they would like to see care delivered in the future?* | Preamble: The pandemic has brought about rapid changes to the way health and social care services are delivered (e.g. more telephone/video consultations). Thinking about these changes:   - How have these changes impacted your care? Have they made it easier/more difficult to get the care you need?   Preamble: Because of the changes that have happened to services during the pandemic, health and social care services are thinking about re-organising they way care is delivered. Thinking about this:   - How would you like your care to be delivered in the future? - In the future, if any changes could be made to health and social care services, what would your ideal care look like? - What do you think needs to happen for this to be possible? - What concerns do you have about your care in the future? |

| **Supplementary Table S2.** Key themes and sub-themes identified within qualitative interviews and free-text questionnaire item responses with illustrative quotes |
| --- |
| **Theme 1: Communication and relationships with healthcare professionals** |
| - *Degree of contact / communication with healthcare professionals*   If I try and ring through to my nurse, before if I rang through to her, she’d pick up the phone or get back to me immediately. But now every time I ring, it’s constantly engaged, you know? I feel bad ringing but I have questions I8 (Female; ≤30; BSRBR-AS)  It would have been good for local medical centre to communicate how they were doing consultations. Q3288 (Female; 31-45; MaMMOTH)   - *Established relationships*   I’ve always had really good honest conversations [with my rheumatology team]. I’ve always known the risks of what I’m taking versus not taking it; how that will impact me longer term; what I need to do if anything every happens. I18 (Female; 31-45; BSR-PsA)   - *Mode of care delivery*   I contacted the GP about six weeks ago about my sore foot, because they were starting to do teleconferencing or video conferencing. And so I thought, “Oh, okay, I’ll try that”, and really she could see my foot but she couldn’t do anything and so it was a bit ineffectual. I1 (Male; 46-64; MaMMOTH) |
| **Theme 2: Transparent, timely and effective pathways of care** |
| - *Expectations about care delivery*   I’ve emailed with a view that a rheumatologist would email me back and a specialist nurse emailed me back, now I know they’re very, very good but the questions I asked I didn’t get an answer to. I4 (Female; 31-45; BSR-PsA)   - *Concerns about changes to accessing/receiving care*   It [*accessing medications*] wasn’t ideal I think because of you had to… you have to go different routes to get your certain medication, and the problem I had then with the hospital team trying to get my (inaudible) which is a biological drug, most of the nurses in the rheumatology unit were allocated to other workstreams because they weren’t doing outpatients, so it was a nightmare getting hold of the hospital and the consultant’s secretary to get the prescriptions done. I12 (Male; 31-45; BSR-PsA)   - *Future organisation of services*   I think broadly they’re likely easier [telephone appointments] in that they’re a quicker point of contact, so you can get through to someone far more quickly than trying to make an appointment than for a face to face meeting. That said, I would hope that at that stage that the healthcare professional would maybe be able to make a quick assessment and understanding of whether they actually needed to see a person, or patient to examine things further. I19 (Male; 46-64; MaMMOTH) |
| **Theme 3: Equitable access to care for everyone** |
| - *Individual challenges to accessing care*   I mean, I’m 73 and I’ve been used to face to face with doctors for 65 years, so that’s a massive change. A lot of people are more reticent on the phone; a lot of people are more outgoing on the phone, so how do they balance that? I5 (Male; 65-74; BSRBR-AS)   - *Concerns about widening inequities*   I feel I am informed, empowered, you know, I’m different to the average lay person in public, so to some degree, I think it’s easier for me to direct my care. We need to establish some help for people, have they got the skills for self-managing? Have they got the skills to recognise when they have concerns? I15 (Male; 46-64; BSRBR-AS)   - *Perceptions of creating more equitable access to services*   I also think increased tele appointments with GPs is a good thing, but we must have good social care in the community to support those vulnerable people who may have attended GP surgeries unnecessarily for reasons of isolation or other social needs Q1417 (Male; 46-64; BSRBR-AS) |
